# Gastric fundus splenosis with hemangioma masquerading as a gastrointestinal stromal tumor in a patient with schistosomiasis and cirrhosis who underwent splenectomy: A case report and literature review

**DOI:** 10.1097/MD.0000000000011461

**Published:** 2018-07-06

**Authors:** Bing Guan, Xiao-Hong Li, Liang Wang, Min Zhou, Zhi-Wu Dong, Guo-Jun Luo, Ling-Ping Meng, Jun Hu, Wei-Yun Jin

**Affiliations:** a Department of Pathology; b Department of Sterile and Supply Center; c Department of Gastroenterology; d Department of Respirology; e Department of Laboratory; f Department of Neurology; g Department of Radiology; h Department of Hepatobiliary Surgery; i Department of Hematologic Oncology, Shanghai 6th People's Hospital Jinshan Branch, Shanghai, PR China.

**Keywords:** gastric fundus, hemangioma, schistosomiasis cirrhosis, splenectomy, splenosis

## Abstract

**Rationale:**

Splenosis is the heterotopic auto-transplantation of the splenic tissues. Gastric splenosis in a rare location mimics a gastrointestinal stromal tumor (GIST). Gastric splenosis with hemangioma has not been reported throughout the literature.

**Patient concerns:**

We report a case of a 74-year-old schistosomiasis cirrhosis splenectomy woman diagnosed with gastric fundus mass. Preoperative computed tomography and endoscopic ultrasonography revealed findings suggestive of a GIST.

**Diagnoses:**

The mass located in the gastric fundus muscularis propria, measuring 3.9 × 2.8 × 2.4 cm with a dark red color, was removed by surgery. In the mass, a 1 × 1-cm red-purple nodule was also found. On microscopic examination, a well-formed splenic tissue divided into two compartments–white pulp and red pulp–separated by an ill-defined interphase known as the marginal zone. However, a nodule in the heterotopic spleen was mainly composed of larger thin-walled muscular vessels. The final diagnosis was gastric splenosis with hemangioma.

**Interventions:**

After discussion in a multidisciplinary conference, the patient was considered for a GIST resection under gastroscopy. In the process of peeling, the surface of the mucosal, submucosal, muscle layers and the tumor surface were diffusely oozing. The effect of electrocoagulation and hemostasis was extremely poor. Therefore, endoscopic surgery was arrested. After dealing with the patient's family, a combination of laparoscopic-gastroscope double-mirror surgery was decided in accordance with the principle of minimally invasive surgery to preserve the stomach. Owing to several adhesions and concealed the location of tumor, we stopped the double-mirror combination surgery plan. Considering the great possibility of a malignant GIST, we still decided to continue the traditional surgical resection. The tumor was then removed via surgery

**Outcomes:**

The patient was favorable with healing and discharged on postoperative day 10.

**Lessons:**

Gastric splenosis with an associated hemangioma is the first well-documented case. Its pathogenesis may be direct implantation. Appropriate medical history taking and Tc-99 m heat-denatured RBC spleen scintigraphy (Tc-99MHDRS) are valuable for its diagnosis; however, pathology is the gold standard. Surgery is a reasonable treatment for gastric splenosis with hemangioma.

## 1 Introduction

Splenosis is the heterotopic auto-transplantation of the splenic tissues arising from traumatic ruptures or iatrogenic splenectomy.^[1]^ Its incidence has been reported to be 67% for traumatic ruptures.^[2]^ Splenosis in the gastric area is a rare location for seeding or spreading. However, gastric splenosis with hemangioma has not been reported throughout the literature. Herein, we report a rare case of a schistosomiasis cirrhosis splenectomy in an asymptomatic elderly woman who had a splenosis with hemangioma located in the gastric fundus wall, which was misdiagnosed as a gastrointestinal stromal tumor (GIST) before on preoperative imaging.

## 2 Case report

A 74-year-old woman was admitted to the Gastroenterology Department of our hospital for an asymptomatic gastric mass. She had a schistosomiasis cirrhosis splenectomy at the age of 29 years.

**Figure 1:**
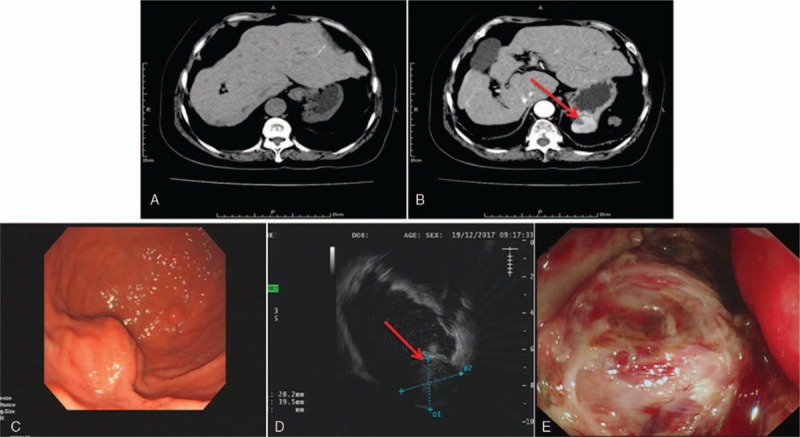
(A) CT scan shows pipe stem cirrhosis. (B) CT scan reveals an approximately 4-cm mass in the gastric fundus wall with a homogeneous contrast enhancement, which is typical in GISTs, and a 1-cm low-density solid lesion with a clear outline within the mass (red arrow). (C) Gastroscopy shows a mass in the gastric fundus. (D) Endoscopic ultrasonography reveals a 3.95 × 2.82-cm slightly low echoic mass with a homogeneous appearance in the muscularis propria of the stomach fundus wall and a low echoic area with a clear boundary in the mass (red arrow). (E) The mucosa and submucosa were cut open, and the mass was exposed in the muscularis propria of the stomach fundus wall. The surface of the mass was covered by larger blood vessels. CT = computed tomography, GIST = gastrointestinal stromal tumor.

The patient was initially submitted to a computed tomography (CT) scan for pneumonia in other hospitals, which revealed pipe stem cirrhosis (Fig. 1A), a well-demarcated 4-cm solid mass confined to the gastric wall suggestive of a GIST (Fig. 1B), and a 1-cm low-density lesion with a clear outline in the mass (Fig. 1B; red arrow). Thereafter, she was submitted to an upper gastrointestinal endoscopy in our hospitals, which revealed a smooth and rounded mass in the gastric wall without mucosal infiltration (Fig. 1C) at the level of the greater curvature. Endoscopic ultrasonography revealed a 3.95 × 2.82-cm slightly low-level echoic homogeneous mass derived from the muscularis propria (Fig. 1D) and a 1 × 1-cm lower level echoic area with a clear boundary in the mass (Fig. 1D; red arrow); these findings confirmed the diagnosis of a gastric GIST. The laboratory test findings were normal, except for the following: platelet count of 369 × 109/L, glutamyl transpeptidase level of 53.4 U/L, total bilirubin level of 22.4 μmol/L, serum creatinine level of 44.0 μmol/L, potassium level of 3.5 mmol/L, and levels of other serum tumor markers (cancer antigen [CA], cytokeratin 19, alpha fetoprotein, carcinoembryonic antigen, CA125, and CA15-3). After discussion in a multidisciplinary conference, the patient was considered for a GIST resection under gastroscopy.

Under the gastroscope, a large submucosal uplift was seen near the posterior wall of the gastric angle. The surface of the mucosa was hyperemic and edematous; the texture was hard; and the activity was poor. After dual-knife labeling, the mucosa and submucosa were opened, and the tumor was initially exposed. The tumor surface was covered with larger blood vessels (Fig. 1E). The IT-NanoKnife was used for detachment around the tumor capsule, and the muscular root penetrated the muscularis propria. In the process of peeling, the surface of the mucosal, submucosal, muscle layers, and the tumor surface were diffusely oozing. The effect of electrocoagulation and hemostasis was extremely poor (taking into account the low coagulation function of liver cirrhosis and the abundant blood supply to the tumor body); further, the procedure took too much time. The tumor roots were poorly exposed owing to persistent oozing. Forcibly removing the full thickness of the stomach wall might lead to difficulties in controlling intra-abdominal bleeding on the serosal side. Therefore, endoscopic surgery was arrested. After dealing with the patient*'*s family, a combination of laparoscopic-gastroscope double-mirror surgery was decided in accordance with the principle of minimally invasive surgery to preserve the stomach. In the process of laparoscopic umbilical puncture point incision, the intestinal mucosa was perforated and was thus subsequently repaired. Owing to the patient*'*s history of 2 abdominal surgeries, several adhesions were seen during laparoscopic surgery, which were then slowly separated. However, the tumor location was high and concealed (gastric angle near the posterior wall); even after following gastroscopy positioning instructions, the tumor still could not be found under laparoscopic direct vision. Therefore, we stopped the double-mirror combination surgery plan. Based on what was seen during the surgery, we communicated with the patient*'*s family again. Considering the great possibility of a malignant GIST, we still decided to continue the traditional surgical resection. The tumor was then removed via surgery; its size was approximately 3.5 × 5 cm, and its blood supply was extremely rich. The abdominal drainage tube and gastrointestinal decompression tube were indwelling. The patient*'*s vital signs were stable; she was then transferred to the intensive care unit and discharged on postoperative day 10.

**Figure 2:**
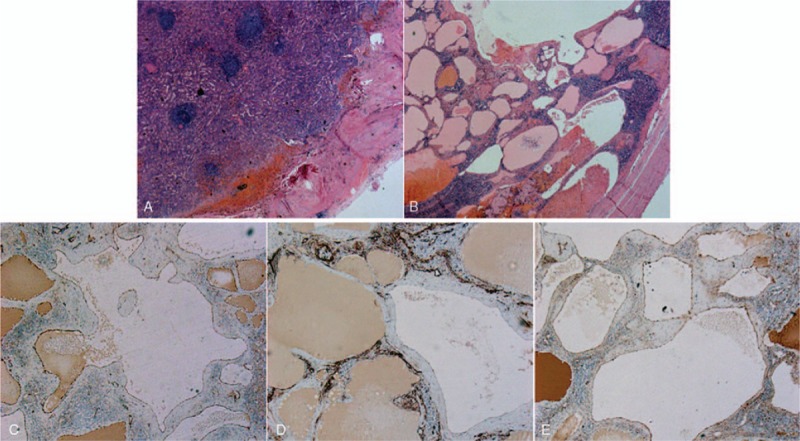
(A) Micrograph shows the same findings as in the normal splenic tissues and the white and red pulps with well-formed splenic corpuscles in the muscularis propria of the stomach fundus wall (H&E, ×40). (B) Micrograph shows a hemangioma in the heterotopic spleen of the stomach fundus wall (H&E, ×40). Endothelial cells of vascular are strongly positive for CD31 (C), CD34 (D), and Factor VIII (E) (×100). H&E = hematoxylin and eosin, CD = cluster of differentiation.

On macroscopic examination, 3.9 × 2.8 × 2.4-cm dark red masses surrounded by a completely thin capsule were observed in the gastric fundus muscularis propria. On the cut surface, the mass appeared red to bluish with scattered white tiny nodules embedded in the muscularis propria. At the edge of the mass, an approximately 1 × 1-cm nodule appearing as a circumscribed, non-encapsulated, honeycomb-like, and red-purple nodule, which formed with dilated congested vascular space with bleeding, was also observed. On microscopic examination, a well-formed splenic tissue divided into 2 compartments—white pulp and red pulp—was separated by an ill-defined interphase known as the marginal zone (Fig. 2A). However, a nodule in the heterotopic spleen was mainly composed of larger thin-walled muscular vessels, which were variably dilated and occasionally displayed thrombosis. The widely dilated vessels showed attenuation of their walls, mimicking a cavernous hemangioma (Fig. 2B). Immuno-phenotypically, the endothelial lining cells of the vascular walls were immunoreactive for cluster of differentiation (CD) 31 (Fig. 2C), CD34 (Fig. 2D), and Factor VIII (Fig. 2E).

The final diagnosis was gastric fundus splenosis with an associated hemangioma.

## 3 Discussion

Splenosis was originally described in 1937 by Shaw and Shaf. However, the term “splenosis,” first used by Buchbinder and Lipkopf in 1939, refers to the dissemination with heterotopic auto-transplantation and implantation of the splenic tissues, which may follow disruption of the spleen*'*s capsule by trauma or iatrogenic splenectomy.^[3]^ Splenosis is commonly found in the abdominal and pelvic cavity, including the greater omentum, serosal surface of the small bowel, parietal peritoneum, mesentery, and diaphragm.^[4]^ Additional potential sites of implantation, including intrathoracic, intragastric, and intrahepatic areas, lungs, kidneys, and brain,^[5–10]^ were reported.

The age of patients with gastric splenosis ranged from 17 to 68 years; the mean age was 44 years, and the median age was 42 years. The interval time from splenectomy to its diagnosis was from 4 to 38 years; the mean interval time was 14.56 years, and the median interval time was 12 years. The size ranged from 1.1 to 5 cm; the mean size was 2.31 cm, and the median size was 2.0 cm. The chief cause of splenectomy related to this condition was traumatic rupture (70%); the other cause was iatrogenic reasons (30%). The main primary suspected diagnosis was GISTs (47%); the other diagnoses included upper gastrointestinal bleeding (17%), gastric mass (12%), gastric smooth muscle tumor (6%; may be GISTs), dyspepsia (6%), gastric splenosis (6%), and gastric band ineffectiveness (6%). The leading method of confirmation was pathology (76%), followed by Tc-99m heat-denatured RBC spleen scintigraphy (Tc-99mHDRS) (12%), cytology (6%), and fine-needle aspiration (FNA) (6%) (Table 1).^[1,2,4–6,11–21]^

**Table 1:**
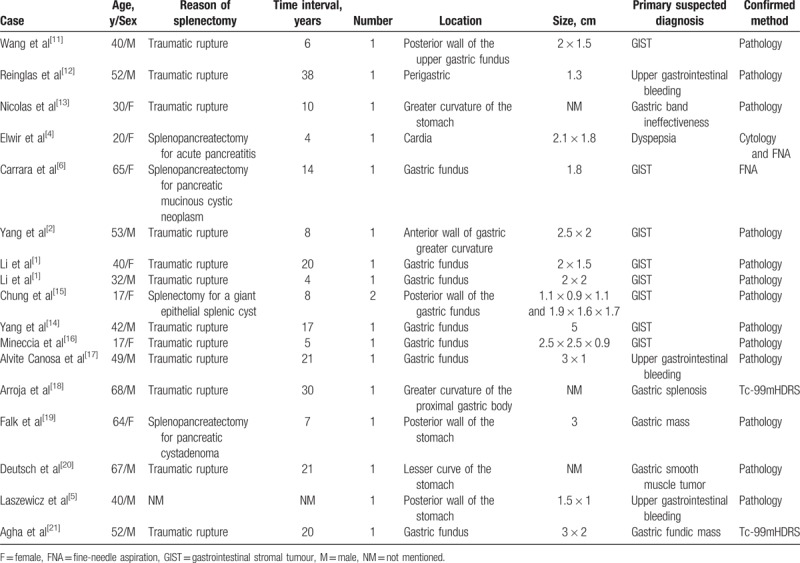
Summary of gastric splenosis case reports.

The pathogenesis of splenosis still remains unclear. Some hypotheses to explain such have been proposed. The seeding/implantation hypothesis in which the isolated splenic pulp may survive after seeding into any position of the abdominal cavity has been demonstrated by animal experiments.^[1]^ However, this hypothesis fails to explain cases found on other sites, such as the liver and lungs. A recent research showed that intra-organ splenosis could spread through the vasculature into the liver and the lungs and that the morphologic and immunologic structures formed in these regenerated autografts were influenced by the organ vasculature and extracellular matrix wherein the tissue fragments settle.^[22]^ Although the hematogenous spread of gastric splenosis is possible, a direct implantation of splenic cells might be caused by needle transfixion during surgical hemostatic maneuvers at the time of emergency splenectomy.^[2]^

Splenosis in the gastric area is an exceptional location for seeding or spreading. To date, no more than 20 cases have been reported. To the best of our knowledge, gastric splenosis with hemangioma has not been reported throughout the literature. It is mainly composed of red and white pulps; however, it does not comprise all kinds of cells in the normal spleen. The implant*'*s or spread spleen*'*s undifferentiated reticular cells are induced to differentiate into the endothelial antrum, capillary vessel, and lymphocytes, which finally create the splenic tissues.^[1]^ Splenic hemangioma is the most common benign neoplasm of the spleen. Splenic hemangiomas are typically solitary lesions and appear as circumscribed, non-encapsulated, honeycomb-like, red-purple masses that frequently blend imperceptibly into the surrounding splenic parenchyma. Microscopically, the majority of splenic hemangiomas are cavernous in nature. A pure capillary architecture is less common, with many lesions containing varying proportions of both cavernous and capillary components. Immuno-phenotypically, splenic hemangiomas show reactivity of the endothelial lining cells of the vascular markers CD31, Von Willebrand factor, Ulex europaeus lectin I, and CD34.^[23]^ Other types of splenic hemangioma include venous hemangioma, benign (infantile) hemangioendothelioma, and diffuse sinusoidal hemangiomatosis.

Gastric splenosis is usually asymptomatic and is only incidentally found in most circumstances. Only in a few cases, patients have upper gastrointestinal bleeding. It is a challenge to diagnose such. Based on the literature statistics, we found that most gastric splenosis cases had been misinterpreted as GISTs. The reason is that conventional ultrasound, CT, and magnetic resonance imaging (MRI) lack typical features to distinguish it from GISTs. Tc-99mHDRS is deemed to be the optimal method for diagnosis of splenosis because it has a higher uptake by the splenic tissues than Tc-99m sulfur colloid scan.^[24]^ Furthermore, superparamagnetic iron oxide-enhanced MRI is a useful diagnostic tool to distinguish gastric splenosis.^[25]^ However, a careful medical history inquiry may provide valuable clues for the differential diagnosis between gastric splenosis and GISTs. On pathology, it is easy to distinguish between the two. Gastric splenosis is mainly composed of red and white pulps. The cells of the red pulp sinusoids are bi-phenotypic immunoreactive for vascular (CD31 and Von Willebrand factor) and histiocytic markers (CD68 and lysozyme).^[23]^ The immunoreactivity for CD8 provides additional evidence for the presence of normal subsets of splenic red pulp lining cells.^[23]^ Most splenic hemangiomas are of cavernous or capillary in nature or of varying proportions of both and have immunoreactivity for vascular markers (CD31, Von Willebrand factor, and CD34). GISTs are mainly composed of spindle cells or epithelioid cells or both in different proportions. The immunoreactivity for CD34 and CD117 discovered on gastrointestinal stromal tumor-1 is helpful for the definitive diagnosis of GISTs. Splenosis is a benign disease and may have some immunologic and splenic-filtering functions, which may be favorable for the organism.^[25]^ Therefore, it is important to establish a correct diagnosis before preoperative imaging to avoid unnecessary surgery, and a thorough follow-up is beneficial in most circumstances. If the patient has clinical symptoms, and a surgery is inevitable, endoscopic or laparoscopy resection or traditional surgery can be selected on the basis of the size or location of the gastric splenosis. If the diagnosis is still unclear, further FNA and cytology examination are recommended.

## 4 Conclusion

In conclusion, gastric fundus splenosis with hemangioma is a unique case with a rare location for seeding or spreading; it is asymptomatic and an incidental finding. It has been misinterpreted as a GIST, and Tc-99mHDRS may be the optimal method for its diagnosis before preoperative imaging. Pathology confirmation is the gold standard. Based on the findings in this case, surgery is a reasonable treatment for gastric fundus splenosis with hemangioma.

## Author contributions

BG performed the pathology and histological examination. XHL, LW, MZ, ZWD, GJL, and LPM assessed the medical records and obtained information on the patient*'*s clinical history. JH and WYJ helped in the final drafting of the manuscript. All authors have read and approved the final manuscript.

Funding acquisition: Bing Guan

Investigation: Bing Guan, Xiao-Hong Li, and Liang Wang

Project administration: Bing Guan

Supervision: Min Zhou, Zhi-Wu Dong, Guo-Jun Luo, and Ling-Ping Meng

Writing **–** original draft: Bing Guan

Writing **–** review and editing: Jun Hu and Wei-Yun Jin

**Data curation:** Bing Guan.

**Funding acquisition:** Bing Guan, Xiao-Hong Li.

**Investigation:** Bing Guan, Xiao-Hong Li, Liang Wang, Min Zhou, Zhi-Wu Dong, Guo-Jun Luo, Ling-Ping Meng, Jun Hu, Wei-Yun Jin.

**Methodology:** Bing Guan, Xiao-Hong Li, Liang Wang, Min Zhou, Zhi-Wu Dong, Guo-Jun Luo, Ling-Ping Meng, Jun Hu, Wei-Yun Jin.

**Software:** Bing Guan.

**Writing – original draft:** Bing Guan.

**Writing – review & editing:** Bing Guan.

## References

[1] Li B Huang Y Chao B Splenosis in gastric fundus mimicking gastrointestinal stromal tumour: a report of two cases and review of the literature. Int J Clin Exp Pathol 2015;8:6566–70. 26261537 PMC4525871

[2] Yang K Chen XZ Liu J Splenosis in gastric wall mimicking gastrointestinal stromal tumour. Endoscopy 2013;45 (suppl 2 UCTN):E82–83. 23526531 10.1055/s-0032-1326263

[3] Buchbinder J Lipkoff C Buchbinder JH Splenosis: multiple peritoneal splenic implants following abdominal injury. A report of a case and review of the literature. Surgery 1939;6:927–34.

[4] Elwir S Thakral B Glessing B Rare subepithelial mass diagnosed as gastric splenosis via EUS-FNA. ACG Case Rep J 2016;3:e101. 27807563 10.14309/crj.2016.74 PMC5062653

[5] Laszewicz W Baniukiewicz A Wroblewski E Upper gastrointestinal hemorrhage secondary to ectopic spleen. Endoscopy 1997;29:56–7. 9083748 10.1055/s-2007-1004072

[6] Carrara S Rahal D Repici A A case of gastric splenosis mimicking a stromal tumour. Clin Gastroenterol Hepatol 2016;14:e50–1. 26505815 10.1016/j.cgh.2015.10.017

[7] Levi Sandri GB Lai Q Bosco S Liver splenosis mimicking hepatocellular carcinoma in cirrhotic liver. Liver Int 2014;34:162. 23834329 10.1111/liv.12250

[8] Sarda R Sproat I Kurtycz DF Pulmonary parenchymal splenosis. Diagn Cytopathol 2001;24:352–5. 11335968 10.1002/dc.1076

[9] Page JB Lenz DL Wong C Right-sided intrarenal splenosis mimicking a renal carcinoma. ScientificWorldJournal 2006;6:2442–4. 17619715 10.1100/tsw.2006.380 PMC5917350

[10] Rickert CH Maasjosthusmann U Probst-Cousin S A unique case of cerebral spleen. Am J Surg Pathol 1998;22:894–6. 9669351 10.1097/00000478-199807000-00011

[11] Wang W Li W Sun Y Intra-gastric ectopic splenic tissue. J Gastrointest Surg 2016;20:218–20. 26438481 10.1007/s11605-015-2940-y

[12] Reinglas J Perdrizet K Ryan SE Splenosis involving the gastric fundus, a rare cause of massive upper gastrointestinal bleeding: a case report and review of the literature. Clin Exp Gastroenterol 2016;9:301–5. 27703390 10.2147/CEG.S91835 PMC5036825

[13] Nicolas G Schoucair R Shimlati R Laparoscopic gastric band removal complicated by splenosis. Clin Case Rep 2016;4:807–11. 27525091 10.1002/ccr3.633 PMC4974435

[14] Yang JB Liu DL Gastric fundus splenosis mimicking stromal tumour. Rev Esp Enferm Dig 2015;107:392–3. 26031875

[15] Chung KM Lau HY Lau WY Ectopic splenic tissues mimicking gastro-intestinal stromal tumour in a patient after splenectomy for a giant epithelial cyst of spleen: a case report. Int J Surg Case Rep 2015;14:13–5. 26204441 10.1016/j.ijscr.2015.06.030 PMC4573406

[16] Mineccia M Ribero D De Rosa G Heterotopic spleen within the gastric wall mimicking a GIST: report of a case. Updates Surg 2013;65:67–70. 22246651 10.1007/s13304-011-0128-x

[17] Alvite Canosa M Castro Ortiz E Alonso Fernandez L Upper gastrointestinal bleeding due to gastric splenosis. Gastroenterol Hepatol 2013;36:59–61. 23218771 10.1016/j.gastrohep.2012.07.006

[18] Arroja B Almeida N Macedo CR Gastric splenosis: a rare cause of digestive bleeding. Rev Esp Enferm Dig 2011;103:377–8. 21770686 10.4321/s1130-01082011000700009

[19] Falk GA Means JR Pryor AD A case of ventral hernia mesh migration with splenosis mimicking a gastric mass. BMJ Case Rep 2009;2009:bcr06.2009.2033. 10.1136/bcr.06.2009.2033 PMC3027525 21954401

[20] Deutsch JC Sandhu IS Lawrence SP Splenosis presenting as an ulcerated gastric mass: endoscopic and endoscopic ultrasonographic imaging. J Clin Gastroenterol 1999;28:266–7. 10192620 10.1097/00004836-199904000-00020

[21] Agha FP Regenerated splenosis masquerading as gastric fundic mass. Am J Gastroenterol 1984;79:576–8. 6611088

[22] Seguchi S Yue F Asanuma K Experimental splenosis in the liver and lung spread through the vasculature. Cell Tissue Res 2015;360:287–96. 25526699 10.1007/s00441-014-2097-0

[23] Kutok JL Fletcher CD Splenic vascular tumours. Semin Diagn Pathol 2003;20:128–39. 12945936 10.1016/s0740-2570(03)00011-x

[24] Fung A Chok K Lo A Hepatobiliary and pancreatic: hepatic splenosis: a rare differential of a liver mass in an HBV endemic area. J Gastroenterol Hepatol 2016;31:1238. 26687675 10.1111/jgh.13275

[25] Wu C Zhang B Chen L Solitary perihepatic splenosis mimicking liver lesion: a case report and literature review. Medicine (Baltimore) 2015;94:e586. 25738479 10.1097/MD.0000000000000586 PMC4553962

